# Biological Therapies in Immune-Mediated Inflammatory Diseases: Can Biosimilars Reduce Access Inequities?

**DOI:** 10.3389/fphar.2019.00279

**Published:** 2019-03-28

**Authors:** Daniel C. Baumgart, Laurent Misery, Sue Naeyaert, Peter C. Taylor

**Affiliations:** ^1^Inflammatory Bowel Disease Unit – Division of Gastroenterology, University of Alberta, Edmonton, AB, Canada; ^2^Department of Gastroenterology and Hepatology, Charité Medical School, Humboldt-University of Berlin, Berlin, Germany; ^3^Department of Dermatology, University Hospital of Brest, Brest, France; ^4^Fresenius Kabi, Eysin, Switzerland; ^5^Botnar Research Centre, Nuffield Department of Orthopaedics, Rheumatology and Musculoskeletal Sciences, Medical Sciences Division, University of Oxford, Oxford, United Kingdom

**Keywords:** rheumatoid arthritis, psoriasis, inflammatory bowel disease, biologic, biosimilar, patient access, pharmacoeconomics, utilization

## Abstract

Biological therapies are an effective treatment for a range of immune-mediated inflammatory diseases (IMIDs), including rheumatoid arthritis, psoriasis, and inflammatory bowel diseases. However, due to their high costs, considerable differences in their utilization exist across the world, even among the various European countries, with many countries restricting access despite professional society guideline recommendations. Adoption of biologics by healthcare providers has been particularly poor in many Central and Eastern European countries. Differences in utilization have also been observed across medical specialties, healthcare providers, and at a regional and national level. The objective of this paper is to provide an overview of the different market access policies for biologics in Europe and to investigate reasons for such differences. One of the potential solutions for providing broader access to IMID patients, where cost is the major barrier, is to encourage the use of biosimilars in place of their reference products. Biosimilars are generally less expensive alternatives to already licensed biological therapies and are approved on the basis that they are similar to the reference product in terms of quality, safety, and efficacy. Budget impact models predict considerable cost savings following the introduction of biosimilars in the next few years. These savings could be used to increase access to biologics and other innovative therapies.

## Introduction

Biological therapies such as anti-tumor necrosis factor (TNF) and anti-interleukin antibodies are widely used for a range of immune-mediated inflammatory diseases (IMIDs), including rheumatoid arthritis (RA) (Smolen et al., 2016), psoriasis (PsO), and psoriatic arthritis (Boehncke and Schon, 2015), Crohn’s disease (CD) (Baumgart and Sandborn, 2012) and ulcerative colitis (UC) (Ordás et al., 2012; Fiorino et al., 2014). Many of these conditions are associated with pain, progressive disability and loss of ability to work (Wolfe and Hawley, 1998; Rapp et al., 1999; Birch and Bhattacharya, 2010; Scott et al., 2010; Tillett et al., 2012; Busch et al., 2014; McWilliams et al., 2014; Carneiro et al., 2017; Husni et al., 2017; Takeshita et al., 2017; Enns et al., 2018; Wu et al., 2018). Furthermore, the prevalence of psychiatric comorbidities such as depression and anxiety can be high in chronic inflammatory diseases (Enns et al., 2018).

The burden these conditions pose to patients and their families cannot be understated; however, there is also a substantial cost to society in terms of long-term treatment costs and decreased work-related productivity (Jacobs et al., 2011; Baumgart and le Claire, 2016; D’Angiolella et al., 2018). The impact of these conditions is further exacerbated by the fact that they disproportionately affect working-age people, with peak onset occurring at 40–50 years of age for RA, 20–40 years of age for UC and CD, and under 40 years of age for PsO (Chorus et al., 2003; Duricova et al., 2014; Queiro et al., 2014).

Since their introduction in the late 1990s, treatment with biologics has transformed outcomes for many patients, with significant improvements in symptoms, work productivity and quality of life being reported (Isaacs et al., 2016). Although all biologics are associated with an elevated infection risk as well as rarer adverse events, the overall benefit-to-risk ratio remains favorable, with data from registry studies providing reassurance as to the long-term safety and tolerability of biologics (van Vollenhoven and Askling, 2005; Raaschou et al., 2013; Garcia-Doval et al., 2017). However, as a result of their high costs and the increasing constraints on healthcare budgets worldwide, differences in the provision of biologics are common and, globally, many patients do not have access to these effective treatments (Putrik et al., 2014; Rencz et al., 2015b; van de Kerkhof et al., 2015; Bergstra et al., 2018). In addition to the costs to the healthcare system, in some European countries patients must make high co-payments to access biologics, which may lead to further inequities in their use (Kawalec et al., 2017).

Biosimilars are alternatives to already licensed biological therapies that are approved on the basis that they are similar to the reference product in terms of quality, safety, and efficacy (Isaacs et al., 2016). To date, multiple studies have demonstrated the efficacy and safety of biosimilars (Cantini and Benucci, 2018; Wiland et al., 2018; Zhao et al., 2018), including the government-funded NOR-SWITCH study (Jorgensen et al., 2017). As they generally have lower acquisition costs than the reference product, biosimilars have been shown to introduce price competition into the market, leading to reduced prices for the treatment of patients (Quintiles IMS, 2017). Consequently, the growing body of evidence obtained from robust development and approval pathways coupled with the budgetary impact of biosimilars has led to health authorities increasing their use of biosimilars (Moorkens et al., 2017).

Reducing healthcare expenditure on biologics and releasing cost savings to support improved access to these important medicines will play a vital role in ensuring that all patients can receive optimal treatment for their disease. An anticipated consequence could be the reduction in long-term complications of the disease as more patients are treated, with associated benefits to the health economy. For example, in the case of RA, registry studies have already demonstrated a dramatic reduction in large joint replacement surgery since the introduction of biologics (Cordtz et al., 2018).

In this article, differences in patient access to biologics for the treatment of RA, PsO and inflammatory bowel diseases (IBD) in Europe will be discussed, including the factors that contribute to disparities in access. The economic burden of IMIDs will also be considered, along with the cost savings already realized or predicted following the introduction of biosimilars. Finally, the potential impact of biosimilars on the provision of more equitable care for all patients will be explored.

## Literature Search

A narrative literature search was conducted in November 2018. The PubMed database was searched using terms related to the disease areas, biologic therapies and patient access. A second search was conducted to identify publications which assessed the impact of biosimilars on patient access and healthcare budgets. The full search strings are provided in Supplementary Table 1. Only articles in English published after 2008 were included. Additional references were identified through searching the bibliographies of retrieved articles.

## Rheumatoid Arthritis

### Guidelines

The European League Against Rheumatism (EULAR) recommends that treatment with a biologic should be considered for patients who have at least one prognostically unfavorable factor, such as early joint damage, and who fail to achieve the treatment target of at least low disease activity (defined by EULAR as a low disease activity state according to any of the validated composite disease activity measures that include joint counts) after 6 months’ treatment with a conventional synthetic disease-modifying anti-rheumatic drug, e.g., methotrexate, with or without glucocorticoids (Smolen et al., 2017).

### Differences in Access to Treatment With Biologics in Europe for RA

Despite the existence of these guidelines, usage of biologics for RA across Western Europe was shown to be highly variable in a series of studies published between 2009 and 2013. In 2009, it was reported that approximately 12% of RA patients were treated with a biologic. However, this varied from almost 30% in Norway, down to less than 5% in Austria (Kobelt and Kasteng, 2009). Similar variation in biologics access was reported in studies by Hoebert et al. (2012) and Laires et al. (2013a). In the Laires et al. (2013a) study, on average 19% of RA patients across 15 Western European countries received a biologic in 2010; this varied from more than 30% in Ireland and Netherlands, down to 7% in Portugal. Similarly, Hoebert et al. (2012) demonstrated large variation in the utilization of TNF inhibitors between four countries (Portugal, Netherlands, Ireland, and Norway) in 2007. In this study, the highest annual utilization rate (calculated as defined daily doses/1,000 inhabitants/day) was reported for Norway (1.89). This was six-fold higher than the rate reported for Portugal (0.32), the country with the lowest utilization rate. In Central and Eastern European countries, biologic usage was consistently lower than that reported in Western Europe. In 2009, less than 5% of RA patients in this region were receiving a biologic. Again, large variations between countries were observed, with 5% usage reported in Hungary, down to less than 1% in Poland (Orlewska et al., 2011).

### Limitations of Biologic Usage Studies

Although these studies clearly highlight the disparities that exist in usage of biologics across Europe, estimates of biologic usage among RA patients who meet the EULAR eligibility criteria for treatment with a biologic were not determined. Instead, biologic usage was typically calculated as a percentage of all RA patients (based on prevalence estimates) (Kobelt and Kasteng, 2009; Orlewska et al., 2011; Laires et al., 2013a) or as a percentage of the total population of the country (Hoebert et al., 2012). In the METEOR registry, usage was calculated only for patients who were being treated by a rheumatologist (Bergstra et al., 2018). It should also be noted that the methodology used to determine the absolute numbers of RA patients being treated with a biologic varied between studies, with data typically being obtained from registries, sales records, insurance claims or a combination of these sources. In addition, patients in Central and Eastern European countries who received biologics as part of clinical trials may not be included in estimates based on insurance claims. As a result of these differences, caution should be applied when performing indirect comparisons between studies.

**FIGURE 1:**
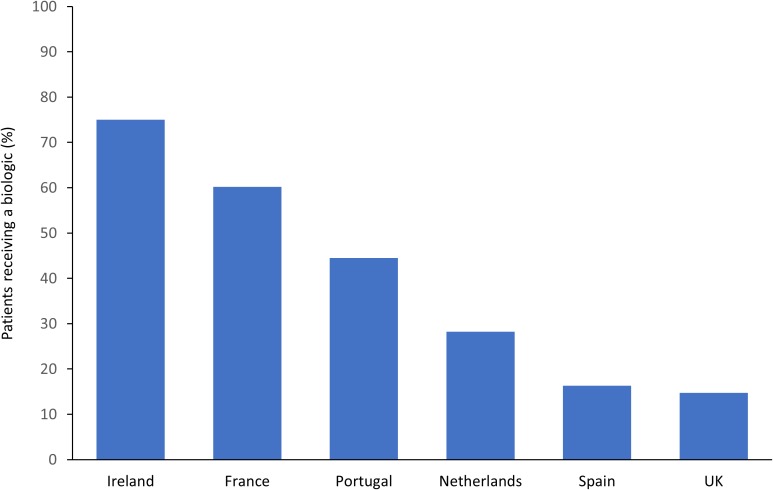
Disparities between European countries in biologic usage in RA patients enrolled in the METEOR registry (Bergstra et al., 2018).

It should also be noted that many of the studies identified in RA are close to a decade old, with few recent studies available. Since then, the market for biologics has continued to expand, with biologics accounting for 27% of all pharmaceutical sales in Europe in 2014 (Remuzat et al., 2017). The market has likely expanded for a number of reasons, including the number of indications approved for each biologic and physicians becoming more confident with their use over time (Pharmaceutical Technology, 2018). Despite this increase, recent data from the METEOR registry has illustrated the large disparity that still exists in the use of biologics as a treatment for RA in Western Europe. In this study, biologic usage amongst RA patients enrolled in this registry varied from 75% in Ireland, down to 16% in Spain, and 15% in the United Kingdom (Figure 1) (Bergstra et al., 2018).

### Impact of Reimbursement Criteria and Macroeconomic Conditions

The reasons for variability in patient access between countries are complex, but differences in national treatment guidelines and reimbursement rules are likely to be key drivers. As biologics are expensive and wide use can significantly impact healthcare budgets, many countries limit access to these therapies through the application of reimbursement criteria that are often stricter than European treatment guidelines, including the EULAR recommendations (Putrik et al., 2014). Reimbursement criteria for biologic therapies are set nationally, or even at the regional or provider level (e.g., hospital), therefore considerable differences can exist between countries and within countries. In a cross-sectional study across 46 European countries, biologics were not reimbursed at all in 10 countries (Putrik et al., 2014). Within the other 36 countries, at least one biologic was reimbursed; however, significant differences in eligibility criteria for the initiation of biologics were reported in terms of disease duration and activity and the need to fail a number of synthetic disease-modifying anti-rheumatic drugs (sDMARDs). For example, out of the 36 countries studied, four require treatment failure with three or more sDMARDs prior to reimbursement, whereas 32 countries require treatment failure with two or fewer sDMARDs. In addition, 11 countries require a moderate disease activity level (Disease Activity Score based on 28 joint count [DAS28] score ≤ 3.2), whereas 20 countries require a higher disease activity level (DAS28 > 3.2). Of note, five countries (Germany, Ireland, Luxemburg, Malta, and Switzerland) had no DAS28 requirement for initiation of a biologic. The impact of national reimbursement criteria on patient access to biologics was evaluated in a population model developed by Kalo et al. (2017). This study estimated that as many as 700,000 RA patients across 39 European countries (including Russia and Turkey) may currently be excluded from biologic treatment despite meeting the eligibility criteria set out by EULAR (Kalo et al., 2017). In this model, the percentage of EULAR-eligible patients who met country-specific guidelines for biologic reimbursement ranged from 86% in countries with less restrictive access to 13% in countries where reimbursement criteria were stricter.

The factors which drive differences in reimbursement criteria between countries, and consequently, patient access to biologics across Europe remain to be fully elucidated. Due to the high direct costs of biologics, differences in cost-effectiveness thresholds that are used in Health Technology Assessments may be a possible explanation, in addition to budget impact assessments. A relationship between gross domestic product (GDP) and reimbursement criteria has been explored by a number of studies, for example Putrik et al. (2014) identified an association between lower socioeconomic welfare and more stringent eligibility criteria for biologics. Similarly, a positive correlation between GDP per capita and patient access to biologics in RA was reported in the METEOR registry and in the study by Laires et al. (2013a) and Bergstra et al. (2018). Macroeconomic conditions, including price and affordability (calculated by comparing relative health care expenditures to the relative price index) were also shown to be a key driver of disparities in access to biologics in Central and Eastern European countries (Orlewska et al., 2011).

### Other Factors Influencing Patient Access to Biologics

On the other hand, in the population model developed by Kalo et al. (2017), only a weak correlation was observed between patient access to biologics and either GDP per capita or percent GDP spent on healthcare. This suggests that other factors are also likely to play a role in determining patient access. In support of this, Laires et al. (2013a) demonstrated in a multivariable regression model using data from 15 European countries, that lower usage of biologics correlated with lower consumption of methotrexate. Furthermore, a relationship between patient access and the biologics distribution channel was also observed, with lower usage of biologics being correlated with a higher proportion of biologics being dispensed in hospitals compared with other settings (Laires et al., 2013a). In a study in Portugal (a country with low biologics usage relative to the European average in 2013), key barriers that limited access to biologics were related to the accessibility of primary healthcare services, difficulties in RA diagnosis among general practitioners, inefficient referral to secondary healthcare, and the controlled process of biologics prescriptions in public hospitals (Laires et al., 2013b). Administrative hurdles and limited numbers of prescribers were also shown to be barriers to access to biologics in Central and Eastern European countries (Orlewska et al., 2011).

### Inequities in Access at a Regional and Individual Level

Use of biologics can also vary substantially within individual countries and at an individual level. For example, in Sweden, a two-fold variation (from 10 to 21%) in biologic penetration between counties at a patient level was reported (Neovius et al., 2011). In the same study, an ecological correlation analysis showed that counties with high sales of biologics tend to initiate treatment in patients with lower c-reactive protein and shorter disease duration. The authors concluded that this warranted further investigation as, despite universal access to treatment in Sweden, different counties seemed to have different initiation thresholds, perhaps reflecting different treatment traditions among rheumatologists as well as county-specific economic considerations. Regional differences were also found in Romania, where urban dwellers and those living in regions with higher living standards were more likely to receive biologics than those living in more deprived areas (Codreanu et al., 2018). This is unlikely to be accounted for by differences in patient incomes, as co-payments for biologics are not required in Romania (Kawalec et al., 2017). Furthermore, in a registry study in Norway, older age and lower education level were associated with reduced access to biologics (Putrik et al., 2016).

## Psoriasis

### Guidelines

The development of targeted and highly effective biological therapies has transformed outcomes for patients affected by moderate-to-severe PsO (Skillen and McKenna, 2018) and their use in other skin diseases such as urticaria and atopic dermatitis is emerging (Saco et al., 2018). Current European guidelines recommend that biologics are used as a second-line treatment for PsO, i.e., if phototherapy and conventional systemic agents, e.g., methotrexate or cyclosporine, were contraindicated, not tolerated, or resulted in an inadequate clinical response (Nast et al., 2015).

### Differences in Access to Biologics in Europe in PsO

**FIGURE 2:**
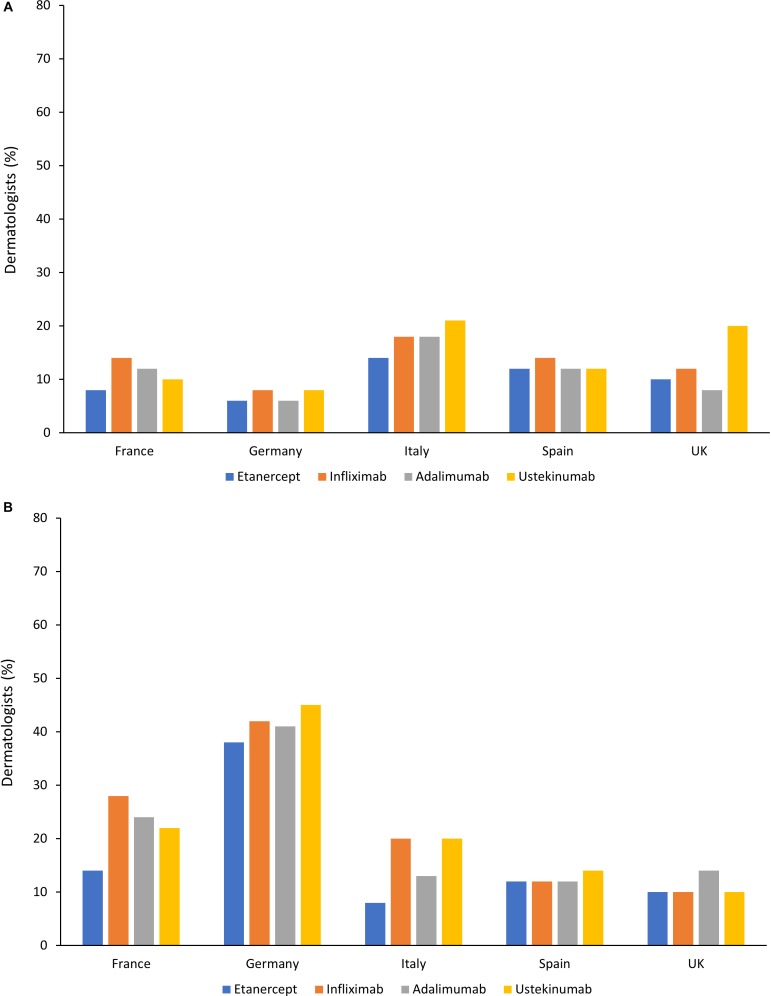
Barriers toward treatment of moderate-to-severe psoriasis with biologics in different countries (Nast et al., 2013). **(A)** Percentage of dermatologists who considered cost to be a strong or very strong barrier to use in patients with moderate-to-severe psoriasis. **(B)** Percentage of dermatologists who considered hospital policies to be a strong or very strong barrier to use in patients with moderate-to-severe psoriasis.

Despite guideline recommendations, biologics are persistently underused for moderate-to-severe PsO and large variations in access to biologics in PsO have been reported, both across and within countries. This underuse of biologics was highlighted in a survey, published in 2015, of European (France, Germany, Italy, Spain, and United Kingdom) and North American dermatologists, which reported that only 20% of patients with moderate-to-severe PsO were receiving treatment with a biologic. Although not all patients with moderate-to-severe PsO need a biologic, the most common reason for dermatologists to not initiate or maintain a biologic was cost (van de Kerkhof et al., 2015). A similar survey of dermatologists in the same European countries as in the previous study, plus Canada, evaluated barriers to the use of systemic therapies in moderate-to-severe PsO. In this survey, 26% of patients were receiving treatment with a biologic. Relevant barriers to biologic use included cost and local hospital/clinic policies. However, as shown in Figure 2, significant differences between countries were reported in terms of the impact of these factors on biologic usage (Nast et al., 2013).

Similar to the situation in RA, access to biologics in PsO appeared to be lower in Central and Eastern European countries compared with Western Europe. This is clearly illustrated in a 2015 study of six Central and Eastern European countries, namely Bulgaria, Croatia, the Czech Republic, Hungary, Poland and Romania. In this study only 0.25% of PsO patients were receiving a biologic therapy. A 15-fold difference in usage was observed between the six countries, with Bulgaria, Croatia, and Poland showing particularly low uptake (Rencz et al., 2015a). Key drivers of these differences were found to be the length of time since a national reimbursement decision was made, number of reimbursed biologics, eligibility criteria in terms of disease severity, and the maximum duration of treatment allowed.

### Differences in Access at a Regional and Individual Level

Inequalities in access to biologics have also been reported at a regional and individual level for PsO treatment. In Sweden, a country typically associated with equal access to healthcare for all residents, a 2.5-fold difference in the rate of initiating a biologic was observed between different geographical regions in 2014 and 2015 (Calara et al., 2017). Another recent study in Sweden demonstrated that increasing patient age was associated with reduced access to biologics for PsO. In this analysis, a Cox proportional hazards model estimated that an increase in age of 30 years corresponded to an estimated 61–68% reduction in the likelihood of initiating a biologic treatment. The reasons for this observation require further investigation, but the authors hypothesized that older patients, particularly if they only have moderate PsO, may be more reluctant to receive systemic treatments. In addition, the authors commented that due to a lack of safety data in older populations, physicians may be reluctant to prescribe biologics to these patients (Geale et al., 2016). Differences in access to biologics at an individual level have also been reported in Italy. In a registry study, less than half (42.5%) of patients starting a systemic therapy for PsO received a biologic. Of note, patients with higher educational attainment and employment status were more likely to receive a biologic than patients considered to have a lower socioeconomic status (Naldi et al., 2017). The factors that contribute to this disparity were unclear, but it was hypothesized by the authors that higher educational and employment status may be associated with better patient negotiating skills or increased empathy from physicians.

## Inflammatory Bowel Diseases

### Guidelines

Biologics have been shown to be effective for the treatment of CD and UC, slowing disease progression, decreasing the need for surgery, and increasing quality of life and work participation (Gulacsi, 2014). Current European guidelines typically recommend that biologics are used as a treatment in IBD for patients with refractory or relapsed disease following initial conventional therapy (Gomollon et al., 2017; Harbord et al., 2017).

### Differences in Access to Biologics in Europe in IBD

**FIGURE 3:**
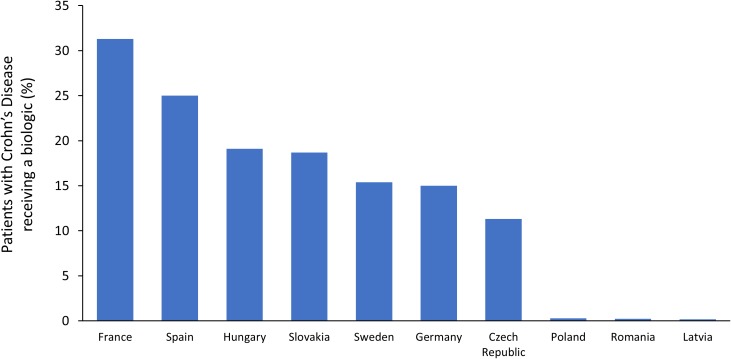
Prevalence of biologic usage in Crohn’s disease across Europe (Pentek et al., 2017).

A systematic review evaluated the patterns of treatment prescribing for CD in Europe (Lelli et al., 2016). Across six individual studies, biologics were used by 8–33% of CD patients, with a trend toward increasing use in recent years. In a questionnaire-based survey in ten European countries, substantial disparities in access to biologics for CD were observed (Pentek et al., 2017). As shown in Figure 3, the prevalence of biologic use was highest in France (31.3% of patients) and lowest in Latvia (0.2% of patients). Uptake of biologics for CD was strongly correlated with GDP per capita and also associated with affordability (drug cost per person as a percentage of GDP). As expected, in countries where biologics are less affordable, eligibility criteria tended to be more restrictive. However, substantial differences were found among countries with similar economic development, suggesting that other factors must play a role in determining patient access to biologics in CD. In Poland, experts identified limited access to IBD centers and to healthcare in general as barriers for access to biologics. In Latvia, the fact that patients must make 25% co-payments for biologics may also have contributed to poor uptake in this country.

Disparities in access to biologics in both UC and CD were evaluated in a study in nine Central and Eastern European countries by Rencz et al. (2015b). The percentage of CD patients treated with biologics ranged from 0.2% in Latvia to 19.1% in Hungary. For UC, biologic utilization rates ranged from 0% in Latvia to 6.4% in Slovakia. In all countries except Romania, lower biologic utilization rates were observed in UC compared with CD, despite the higher prevalence of UC. A possible explanation provided by the authors is that biologics for UC were only approved 8 years after biologics for CD. Within this study, great heterogeneity (up to 96-fold for CD) was found in access to biologics across the countries, with Bulgaria, Poland, Romania, and the Baltic States having lower access than the Czech Republic, Hungary, and Slovakia. This difference could not be explained by epidemiological factors, drug prices or total health expenditure. It was hypothesized that health deterioration linked to IBD might be valued differently against other systemic inflammatory conditions in certain countries, and this may contribute to variations in utilization of biologics for IBD.

## Economic Impact of Immune-Mediated Inflammatory Diseases

### Rheumatoid Arthritis

In 2006, it was estimated that in Europe there were approximately three million patients with RA and that the annual total economic burden of RA was €45 billion. This amounted to approximately €13,500 per patient per year, with Germany reporting the highest costs of €22,500 per patient per year. In this study, only 14% of the total annual cost was spent on drug costs; more than half of all costs related to production losses (loss of work capacity) and informal care (Lundkvist et al., 2008). Similar findings were reported by the National Rheumatoid Arthritis Society in the United Kingdom in 2010 (National Rheumatoid Arthritis Society, 2010). In this report, NHS expenditure for RA was approximately £700 million per year; however, the overall cost to the United Kingdom economy due to productivity losses was estimated to be almost £8 billion per year. A more recent study in Italy estimated the total economic burden associated with RA to be €2 billion per year, with 45% (€0.9 billion) due to indirect costs (Mennini et al., 2017). The high indirect costs reported in these studies are driven by the fact that RA is frequently diagnosed in working age individuals and because almost one-third of RA patients ultimately give up work as a result of their condition (National Rheumatoid Arthritis Society, 2010).

### Psoriasis

For PsO, a systematic review from 2014 reported annual total (including direct and indirect) costs per patient of €11,928 in Sweden, €8,372 in Italy, and up to €6,707 in Germany (Feldman et al., 2014).

### Inflammatory Bowel Disease

The total direct costs in Europe for IBD have been estimated to be as high as €5.6 billion per year (Burisch et al., 2013). Other estimates for economic burden in Europe are €12.5–29.1 billion for UC and €2.1–16.7 billion for CD annually (Yu et al., 2008; Cohen et al., 2010). As IBD affects young people, indirect costs resulting from loss of productivity can be substantial. In a review of studies from Europe and North America, indirect costs of up to $14,500 and $6,500 per year per patient were reported for CD and UC, respectively (Kawalec, 2016).

### Impact of Biologics on Indirect and Other Medical Costs

Although the introduction of biologics has increased direct healthcare costs for IMIDs, their potential impact on indirect costs and on other medical costs, such as hospitalization and surgery, is also an important consideration. For example, use of biologics can reduce the rate of expensive surgical interventions in RA and IBD (Moura et al., 2015; Chen et al., 2017; Mao et al., 2017). Furthermore, the improved quality of life associated with biologic therapy can increase productivity and reduce the societal impact of RA (Strand and Singh, 2010), and earlier access to biologics for RA patients has been shown to have a positive effect on employment status (Olofsson et al., 2017). Similarly, improvements in the severity of PsO have been associated with increased work-place productivity and a reduction in indirect costs (Graham et al., 2015). For IBD, treatment with biologics has been associated with improvements in work-related productivity (Busch et al., 2014).

### Impact of Biosimilars

Biosimilars are cost-effective alternatives to their reference biologic products, providing an opportunity to expand access to biologics. Regulatory approval of biosimilars in Europe is conducted by the European Medicines Agency (EMA). The regulatory pathway permits biosimilar manufacturers to build on the safety and efficacy experience of the reference product, without the need for extensive clinical trials (European Medicines Agency [EMA], 2017). However, despite the overall abbreviated regulatory process, similarity between a reference product and its biosimilar must be determined by a rigorous three-tier process. The first tier involves extensive *in vitro* analytical physicochemical and functional studies to comprehensively compare the structure and biological function of the two drugs. In the second tier, pharmacodynamic studies compare drug interactions with downstream physiological targets in relevant *in vivo* models. In the third tier, comparative clinical studies are performed to compare pharmacokinetics, pharmacodynamics and safety, including immunogenicity, in healthy volunteers. This is followed by comparative randomized clinical trials in sensitive patient populations with appropriate endpoints to demonstrate similarity in terms of efficacy, safety, and immunogenicity (European Medicines Agency [EMA], 2017). Following regulatory approval, continued pharmacovigilance is essential for monitoring the ongoing safety profile all new drugs, including biosimilars (Danese et al., 2017; European Medicines Agency [EMA], 2017; Araújo et al., 2018; Kay et al., 2018).

Once a biosimilar is approved it can be prescribed to treatment naïve patients or to patients who have previously received treatment with the reference product. While robust regulation ensures biosimilarity of the medicine, there is currently a lack of data on the potential impact of multiple switches between biosimilars and the reference product (Cohen et al., 2018). Another issue associated with switching is the loss of efficacy or occurrence of an adverse event that cannot be explained based on the known pharmacology of the drug. This phenomenon, termed the ‘nocebo effect,’ is not specific to biosimilars, but can occur whenever a patient has a negative perception of a therapy (Planès et al., 2016; Rezk and Pieper, 2018). This can reduce medication adherence or even result in patients switching back to the reference product (Boone et al., 2018). Indeed, in biosimilar studies a possible nocebo effect has been demonstrated by an increase in discontinuation rates in open-label studies relative to double-blind studies (Odinet et al., 2018). In order to reduce the impact of any nocebo affect, appropriate patient education will be crucial to build confidence and increase understanding of biosimilars among patients. Finally, any additional resources required to facilitate a switch from a reference product to a biosimilar, e.g., additional outpatient appointments, may partially off-set the cost savings associated with the biosimilar in the short term (Barnes et al., 2018). However, of note, a Danish registry study found no evidence of an increase in outpatient visits following a switch from reference infliximab to a biosimilar (Glintborg et al., 2018). In addition, pharmacovigilance is needed for all drugs and the need to monitor multiple biosimilar products may require pharmacovigilance systems to adapt to handle increasing amounts of data (Azevedo et al., 2017).

Since 2013, biosimilars for infliximab, then etanercept and finally adalimumab have been approved and launched for the treatment of IMIDs. Additional adalimumab biosimilars are expected to be launched shortly. The economic benefits of the introduction of these biosimilars can be seen across Europe. For example, through a national tender process, Norway negotiated a price reduction of 69% for biosimilar infliximab compared to the reference product (Matusewicz et al., 2015). Furthermore, in the United Kingdom, the introduction of infliximab and etanercept biosimilars resulted in cost savings of £38.8 million over 2 years (2015–2017), with the reference products also reducing their prices in response to the availability of lower priced biosimilars. Nevertheless, in this market, the introduction of biosimilars led to significant reductions in the utilization of branded infliximab (Remicade^®^) and etanercept (Enbrel^®^) (Aladul et al., 2017).

Looking to the future, a budget impact model published in 2018 has estimated the cost savings expected to be achieved once adalimumab, infliximab, and etanercept biosimilars are all available in the United Kingdom (Aladul et al., 2018). This model predicted that infliximab and etanercept biosimilars would replace their corresponding reference agents by 2020. Adalimumab biosimilars were predicted to take 19% of the rheumatology and gastroenterology market by 2020. Based on the introduction of these biosimilars, a reduction in expenditure of £44 million on biologics over the next 3 years was estimated. In another model assessing the budget impact of an infliximab biosimilar, the annual projected cost savings were estimated to range from €25.79 million (assuming a 10% price reduction) to €77.37 million (30% price reduction) across Belgium, Germany, Italy, Netherlands, and the United Kingdom (Jha et al., 2015). A similar model estimated that the introduction of an etanercept biosimilar would save the National Health Service in Italy €90 million over 5 years (Ravasio et al., 2018). In CD, the total potential cost savings achievable over 3 years after the introduction of an infliximab biosimilar in Bulgaria, the Czech Republic, Hungary, Poland, Romania, and Slovakia according to a budget impact model, were expected to be between €8 million (if switching was not allowed) up to €16.9 million (if switching was allowed in 80% of the patients) (Brodszky et al., 2016). Following the recent launch of four adalimumab biosimilars, even greater cost savings are anticipated, with NHS England now estimating that £300 million will be saved by 2021 (NHS England, 2018).

Cost savings from biosimilar uptake can be used to either improve access to biological treatments for patients qualifying for this treatment in countries where access is limited, or the savings can be allocated to other diseases or areas of care, thus generating incentives for wider use and prescription of cost-effective biosimilars instead of reference products. An example of this can be found in Italy, where a number of regions have introduced a system whereby 50% of the savings generated from biosimilar uptake are reallocated to augment by 20% the budget dedicated for coverage of innovative medicines (IMS Institute for Healthcare Informatics, 2016). There is also a need to ensure access to the latest treatments for patients while balancing the need for affordable drugs and health care sustainability. However, existing patent protections for new drugs should continue to incentivize innovation in the years to come.

### Benefits of Biosimilars From the Patient, Healthcare Provider, and Payer Perspective

The cost savings from biosimilars can improve patient access by broadening national reimbursement criteria to reach parity with European clinical guidelines. For both patients and healthcare providers this may offer the benefit of improved clinical outcomes. For example, IMID patients with early or moderate disease have been shown to benefit from access to biologics, as demonstrated by improvements in clinical and functional outcomes (St Clair et al., 2004; D’Haens et al., 2008; Emery et al., 2008; Colombel et al., 2010, 2018; Baranauskaite et al., 2012; Atsumi et al., 2017; Kavanaugh et al., 2017). Although initially wary of biosimilars, patients have more recently become more amenable to switching, at least in part due to an understanding of the wider societal benefits of biosimilars.

From a payer perspective, the potential short-term cost savings and expanded patient access as a result of introducing biosimilars could also translate into long-term savings, as treating patients with biologics at an earlier disease stage may reduce long-term costs by, for example, delaying or avoiding costly hospitalizations and surgical interventions (Moura et al., 2015; Mao et al., 2017).

### Future Approaches to Improve Biologic Access and Uptake

In order to increase the uptake of biosimilars, any preconceived negative physician or patient perceptions will need to be addressed as well as any reluctance to prescribe. This will require raising awareness of biosimilars and providing education to healthcare professionals on the robustness of the development and regulatory requirements for biosimilars and also their safe use in different indications. As an example, medical professionals were initially concerned about extrapolation, namely the lack of clinical data in IBD for the infliximab biosimilar CT-P13, since clinical trials were conducted in ankylosing spondylitis and RA indications only. There were also concerns about increased immunogenicity related to post-translational modification of the protein. However, data from real-world use have helped to confirm the similarity between CT-P13 and the reference product in terms of efficacy, safety and immunogenicity in IBD, although further studies on switching between products may be useful (e.g., cross-switching between biosimilars) (Gecse et al., 2016; Gabbani et al., 2017; Kurti et al., 2018).

Medical societies and healthcare professionals can provide advocacy and education for biosimilars and have issued scientific reviews and/or position papers. For example, the International Psoriasis Council has provided a global overview of the regulations, uptake and potential implications of the use of biosimilars for dermatology in clinical practice (Cohen et al., 2017). The Council recommends that dermatologists should take an active role in the development of biosimilar-prescribing policies within their respective healthcare settings and with the support of government agencies.

In addition to increasing biosimilar uptake, other issues will need to be addressed in order to improve access to biologics generally. While these issues may be country specific, they include streamlining the infrastructure for distributing biologics, optimizing the referral pathways for patients eligible for biologics, and easing the restrictions on who can prescribe biologics. For example, the involvement of nurse specialists who can take responsibility for such activities as screening, patient education, prescription coordination for home drug delivery, monitoring and data collection, could facilitate the wider use of biologics for the treatment of IMIDs (Palmer and El Miedany, 2010).

### International Recommendations for Cost-Effective Use of Biologics

The benefits of taking a more cost-effective approach to the usage of biologics in the treatment of IMIDs is now reflected in international guidelines, such as those provided by EULAR, which state that cost-effective treatment approaches must be preferred as long as safety and outcomes are similar and in line with therapeutic paradigms when choosing a treatment strategy for RA (Smolen et al., 2017). Similar advice is now being given to physicians by individual countries. For example, in the United Kingdom, the National Institute for Health and Clinical Excellence has given guidance to rheumatologists and gastroenterologists that treatment for RA and UC should be initiated with the least expensive biologic (NICE, 2015, 2016). Treatment costs are also a consideration in the guidance issued by the French Society for Rheumatology, which states that treatment decisions for RA should be based chiefly on efficacy and safety data, while also factoring in the costs of management (Gaujoux-Viala et al., 2014).

## Conclusion

Across Europe differences in patient access to biologic therapies have been reported. Factors which contribute to these differences are complex and can be country-specific; however, macroeconomic conditions, including drug cost per person as a percentage of GDP, have frequently been shown to be a key driver of disparities in the usage of biologics. As a result, access to high-cost biologic treatments are often particularly poor in low-income countries. Continued research is necessary to ensure that payors and patient groups have access to the data they need to fully understand the costs and benefits of adopting biosimilars. Of note, there is a general lack of long-term data for all countries and the extent to which the introduction of biosimilars will increase uptake of biologics, including at an earlier disease stage, remains to be fully determined. As registries continue to accumulate real-world data, it will be possible to answer these questions, as well as longer-term questions relating to the health economic implications and wider societal benefits following the introduction of this generation of biosimilars. Other gaps in the literature include how best to communicate the relevant issues to patients when a switch from a reference product to a biosimilar is proposed, and how integrated use of biosimilars in clinical practice will impact the treatment of IMIDs.

Several biosimilars are now available for patients with IMIDs. These offer the potential to reduce acquisition costs of biologics and may lead to savings in other areas, for example, through a reduced need for costly procedures and hospitalizations in patients who have benefited from earlier access to biologics. This may allow more patients who are eligible for biologics to be treated with these effective medications, or to release budgets that can be reallocated to other disease areas or services. It is hoped that the introduction of biosimilars will contribute to reducing current inequities in the use of biologic treatments and potentially reduce the economic and social burden associated with IMIDs.

## Author Contributions

All authors participated in the selection and interpretation of the literature reviewed, wrote or critically reviewed the manuscript, and reviewed and approved the final version.

## Conflict of Interest Statement

DB: Served over the past 15 years on external scientific advisory boards for 4d Pharma, Abbott, AbbVie, Allergan, Amgen, Astra Zeneca, Bayer, Biogen, Boehringer Ingelheim, Bristol-Myers Squibb (BMS), Celgene, Cellerix, Centocor, CSL Behring, Dr. Falk, Elan, Eli Lilly, Ferring, Forward, Genentech, Gilead, Glaxo Smith Kline (GSK), Hitachi, Janssen, Johnson & Johnson, Merck, Merck Serono, Merck Sharp Dohme (MSD), Millenium, Novartis, Novo Nordisk, Ocera, Otsuka, PDL Biopharma, Pfizer, Prometheus, Recordati, Roche, Sandoz, Sanofi Aventis, Schering, Schering-Plough, Shield, Shire, Takeda, Theravance, TiGenix, Tilliotts Pharma AG, UCB Pharma, Vifor. All of his activities and contracts are in conformity with the “FSA-Kodex Fachkreise” (voluntary self-monitoring code for expert consultants to the pharmaceutical industry), have been checked and approved by the Faculty of Medicine. LM: AbbVie, Amgen, Biogen, Fresenius Kabi, Janssen-Cilag, Leo Pharma, Lilly, MSD, Novartis, Pfizer, Sanofi, UCB. SN: Consultant for Fresenius Kabi SwissBioSim. PT: AbbVie, Biogen, Celgene, Fresenius Kabi, Galapagos, Gilead, GSK, Janssen, Lilly, Novartis, Pfizer, Roche, Sandoz, Sanofi, UCB.
