# Supplementary material for: Biological Therapies in Immune-Mediated Inflammatory Diseases: Can Biosimilars Reduce Access Inequities?
Source: Front Pharmacol. 2019 Mar 28;10:279. doi: 10.3389/fphar.2019.00279 (PMC6447826; doi:10.3389/fphar.2019.00279)
Supplement: Supplementary file 1 [file Table_1.DOCX]

# Supplementary Table 1

# Search 1. Inequities in access to biologics

| Search | Search string |
| --- | --- |
| #1 | Immune mediated inflammatory disease* OR rheumatoid arthritis OR RA OR psoriasis OR psoriatic arthritis OR inflammatory bowel disease OR Crohn* OR ulcerative colitis OR rheumatol* OR dermatol* |
| #2 | Biologic* OR bDMARD OR tumour necrosis factor OR interleukin OR integrin OR JAK OR T-cell OR B-cell OR rituximab OR anakinra OR tocilizumab OR sarilumab OR ustekinumab OR secukinumab OR ixekizumab OR brodalumab OR guselkumab OR vedolizumab OR tofacitinib OR baricitinib OR abatacept OR adalimumab OR certolizumab OR etanercept OR golimumab OR infliximab |
| #3 | treatment pattern OR reimburse* OR inequit* OR access |
| #4 | #1 AND #2 AND #3 |
| #5 | Limited to Title/Abstract, Publication Year 2008-2018, English Language |

# Search 2. Budget impact and cost savings for biosimilars

| Search | Search string |
| --- | --- |
| #1 | Immune mediated inflammatory disease* OR rheumatoid arthritis OR RA OR psoriasis OR psoriatic arthritis OR inflammatory bowel disease OR Crohn* OR ulcerative colitis OR rheumatol* OR dermatol* |
| #2 | biosimilar |
| #3 | Cost OR budget OR saving OR access |
| #4 | #1 AND #2 AND #3 |
| #5 | Limited to Title/Abstract, Publication Year 2008-2018, English Language |
